# Genome Sequence of *Lactobacillus fabifermentans* Strain T30PCM01, Isolated from Fermenting Grape Marc

**DOI:** 10.1128/genomeA.00060-14

**Published:** 2014-02-20

**Authors:** Laura Treu, Veronica Vendramin, Barbara Bovo, Alessio Giacomini, Viviana Corich, Stefano Campanaro

**Affiliations:** a Department of Agronomy, Food, Natural Resources, Animals and Environment (DAFNAE), University of Padova, Padua, Italy; b Department of Biology, University of Padova, Padua, Italy

## Abstract

Here, we report the draft genome assembly of *Lactobacillus fabifermentans* strain T30PCM01 isolated from grape marc. Its genome is the largest (3.58 Mbp) among *Lactobacillus* species and reveals an enormous potential for carbohydrate utilization and transcriptional regulation.

## GENOME ANNOUNCEMENT

*Lactobacillus* is the most diverse genus among lactic acid bacteria (LAB), a group of Gram-positive bacteria that produce lactic acid as their major fermented end product and that are often involved in food fermentation (1, 2). Only recently, *Lactobacillus fabifermentans* was proposed as a new species and was associated with spontaneous cocoa bean fermentation (3). Some strains of this species (including *L. fabifermentans* T30PCM01) were isolated in northeast Italy from grape marc collected after a prolonged storage period to allow spontaneous alcoholic fermentation (4, 5). Grape marc is the main solid by-product of the wine industry in Mediterranean countries, with this raw residue occurring in a quantity of about 10 million tons per year (6). After alcoholic fermentation, grape marc is used in Mediterranean countries in the production of traditional alcoholic beverages (like the Italian grappa). To better understand the ability of *L. fabifermentans* to grow in this fermented by-product characterized by extreme environmental conditions (7), we have sequenced and annotated the genome of *L. fabifermentans* T30PCM01.

Sequencing was performed by the Ramaciotti Centre, Sydney, Australia, using a MiSeq sequencer (Illumina) that produced 1,413,271 paired-end reads of 250 + 250 bases. Libraries were produced with the Nextera XT kit (Illumina) that gave an insert size between ~350 bp and 1.5 kbp. Short reads were assembled with ABySS 1.3.6 (8) (*k*-mer, 63) obtaining 94 scaffolds >1 kb, for a total of 3,580,413 bp, with a mean G+C content of 44.9%. A second assembly was performed with Velvet 1.2.10 (9) (*k*-mer, 131) obtaining 149 scaffolds >1 kbp. The two assemblies were aligned using Mauve (10) to verify if some gaps were specifically resolved by one of the two assemblers. The scaffold order (from scaffold 01 to scaffold 94) was determined by aligning them against the genomes of some closely related species (*Lactobacillus pentosus* KCA1*, Lactobacillus plantarum* strains JDM1, NC8, P8, WCFS1, and ZJ316, and *L. plantarum* subsp. *plantarum* ATCC 14917).

Gene prediction and annotation were performed with RAST (11), obtaining 3,749 genes, 3,437 of which are protein coding. An independent gene finding and annotation process was performed using GeneMark.hmm 2.8 trained on *L. plantarum* (12), and with BASys (13), obtaining 3,502 protein-coding genes, 94.4% of which (3,306/3,502) were also identified with RAST (11). Analysis of the subsystem category distribution performed with RAST (11) revealed that the categories of carbohydrates, phosphorus metabolism, sulfur metabolism, membrane transport, and cell wall and capsule contain the highest number of functional roles among the *Lactobacillus* species that are completely sequenced and present in the RAST database (443, 46, 11, 59, and 146, respectively). The regulation and cell signaling category comprises 42 functional roles, a large number in comparison to other *Lactobacillus* species, suggesting a high regulatory potential.

### Nucleotide sequence accession number.

The whole-genome shotgun project of *L. fabifermentans* T30PCM01 has been deposited at DDBJ/EMBL/GenBank under the accession no. AWWK00000000.
